# Fungal Rhinosinusitis: Prevalence and Spectrum in Singapore

**DOI:** 10.7759/cureus.7587

**Published:** 2020-04-08

**Authors:** Nada A Alshaikh, Khalid S Alshiha, Samuel Yeak, Stephen Lo

**Affiliations:** 1 Otolaryngology, Dammam Medical Complex, Dammam, SAU; 2 Otolaryngology, King Fahad University Hospital, Khobar, SAU; 3 Otolaryngology, Tan Tock Seng Hospital, Singapore, SGP

**Keywords:** fungal rhino sinusitis, prevalence, spectrum, singapore

## Abstract

Background

Fungal involvement of the paranasal sinuses has been described more than two centuries ago. In the current article, it is referred to as fungal rhinosinusitis (FRS) which is a general term that is used to describe a spectrum of pathologically, immunologically, and clinically different disease entities affecting the paranasal sinuses where fungus is thought to be the major potential etiology.

Objective

To determine the incidence and spectrum of FRS in Singapore and to compare our findings with international figures through literature review.

Methods

A retrospective review of the clinical charts, radiological and laboratory results, and operative reports of all patients who underwent endoscopic sinus surgery at an ENT department of a tertiary referral hospital in Singapore over five-year period.

Results

Out of 533 functional endoscopic sinus surgeries performed during the period of the study for management of chronic rhinosinusitis, 44 (8.4%) were found to fit the criteria for diagnosis of FRS. Twenty (45.5%) were eosinophilic FRS and 24 (54.5%) were fungal balls. Invasive FRS has not been encountered. Clinical presentation, investigations, and management of both groups of patients are discussed.

Conclusion

Fungal rhinosinusitis is not uncommon in Singapore. Fungal ball and eosinophilic mucin fungal rhinosinusitis are among the most common forms encountered in this part of the world.

## Introduction

Fungal rhinosinusitis (FRS) is not uncommon form of sinonasal pathology [1]. It was first described by Plaignaud in 1791 when he reported a case of maxillary fungal sinusitis [2]. However, it was not until nearly the last three decades when a tremendous interest in understanding the pathology by which the different underlying mechanisms of FRS evolved [3]. Currently, FRS is referred to as a spectrum (and not a continuum) of distinct fungal-related sinus diseases with discrete pathologic features that does not seem to undergo transition from one condition to another [1].

Despite improvement in laboratory technology, diagnostic tools, and understanding of fungal-related rhinosinusitis, the exact underlying pathophysiology that leads to the development of one form of the disease over another in different patients is not fully understood and yet to be determined [1-4].

FRS is classified into different forms according to several factors such as the immune status of the patient and the distinctive clinical and radiological features of each form [1,4,5]. Histopathological classification of FRS into invasive and non-invasive forms is believed to be the most acceptable and clinically applicable method of classification. It relies on histological identification of fungal elements within the host tissue in order to define invasiveness [5]. Based on such classification, non-invasive FRS is referred to those fungal-related sinus conditions which lack histological confirmation of fungal tissue invasion, yet present with a distinctive pathological and clinical features differentiating them from one another. Under this form of non-invasive fungal rhinosinusitis comes three discrete pathologies, namely, eosinophilic mucin fungal rhinosinusitis (EMFRS) which can be either allergic in nature (Allergic fungal rhinosinusitis) or non-allergic type which is characterized by identification of fungus and fungal mucin within the sinuses with histological confirmation of tissue eosinophilia and absence of atopy, fungal ball (FB) - inaccurately called Mycetoma - and saprophytic fungal infestation (SFI) [5-6]. The invasive fungal rhinosinusitis, on the other hand, includes acute invasive fungal rhinosinusitis (AIFRS), chronic invasive fungal rhinosinusitis (CIFRS), and granulomatous invasive fungal rhinosinusitis (GIFRS) [7]. The fact that treatment and prognosis vary considerably among different subtypes of FRS, making an accurate diagnosis is of paramount value as a guide in establishing the appropriate management plan [4,8-9].

Certain types of fungal rhinosinusitis have a distinctive geographical distribution [4]. Allergic fungal rhinosinusitis, for example, is more commonly found in the Southeast and Mississippi basin of the United States and also frequently found in different parts of Saudi Arabia [9-11].

The aim of this pilot study is to find out the incidence and spectrum of fungal rhinosinusitis in Singapore which have never been looked at before. This along with clinical presentations, diagnosis, and management of FRS is discussed in comparison with the international data through literature review.

## Materials and methods

This study was conducted at a tertiary referral university hospital in Singapore and the protocol was approved by the local research ethics committee and review board. Retrospective medical charts review of all patients who underwent endoscopic sinus surgery (ESS) at our ENT department between January 2004 and June 2009 was performed. Demographic data, clinical presentation, computed tomography (CT) scan findings, operative notes, histopathology report, and microbiological cultures and/or stains were all examined for patients with diagnosis of sinusitis/rhinosinusitis.

To identify all spectrum of cases of fungal rhinosinusitis among all rhinosinusitis patients that were treated with ESS, Bent and Kuhn criteria for AFRS and the diagnostic criteria of IFRS and FB proposed by de Shazo and colleagues were adopted [7,12]. Presence of one or more of these criteria was an indication for further detailed analysis of the medical chart in order to ensure that fungal rhinosinusitis was neither incidentally over-diagnosed nor overlooked. Furthermore, the immune status of the patients along with the clinical presentation, evidence of fungal tissue invasion in histology, and disease progression was also considered in making the final diagnosis.

## Results

Five hundred and ninety-one endoscopic sinus and skull base surgeries were performed during the study period. Fifty-eight cases were diagnosed as tumors of the nose, paranasal sinuses, or skull base and thus were excluded. Therefore, a total of 533 endoscopic sinus surgeries performed for management of rhinosinusitis were reviewed. Amongst these cases, 44 (8.26%) were found to match the criteria of fungal rhinosinusitis of which 24 (54.5%) were FB and 20 (45.5%) were EMFRS (AFRS = 5, EFRS = 15).

Demographic data

**Figure 1:**
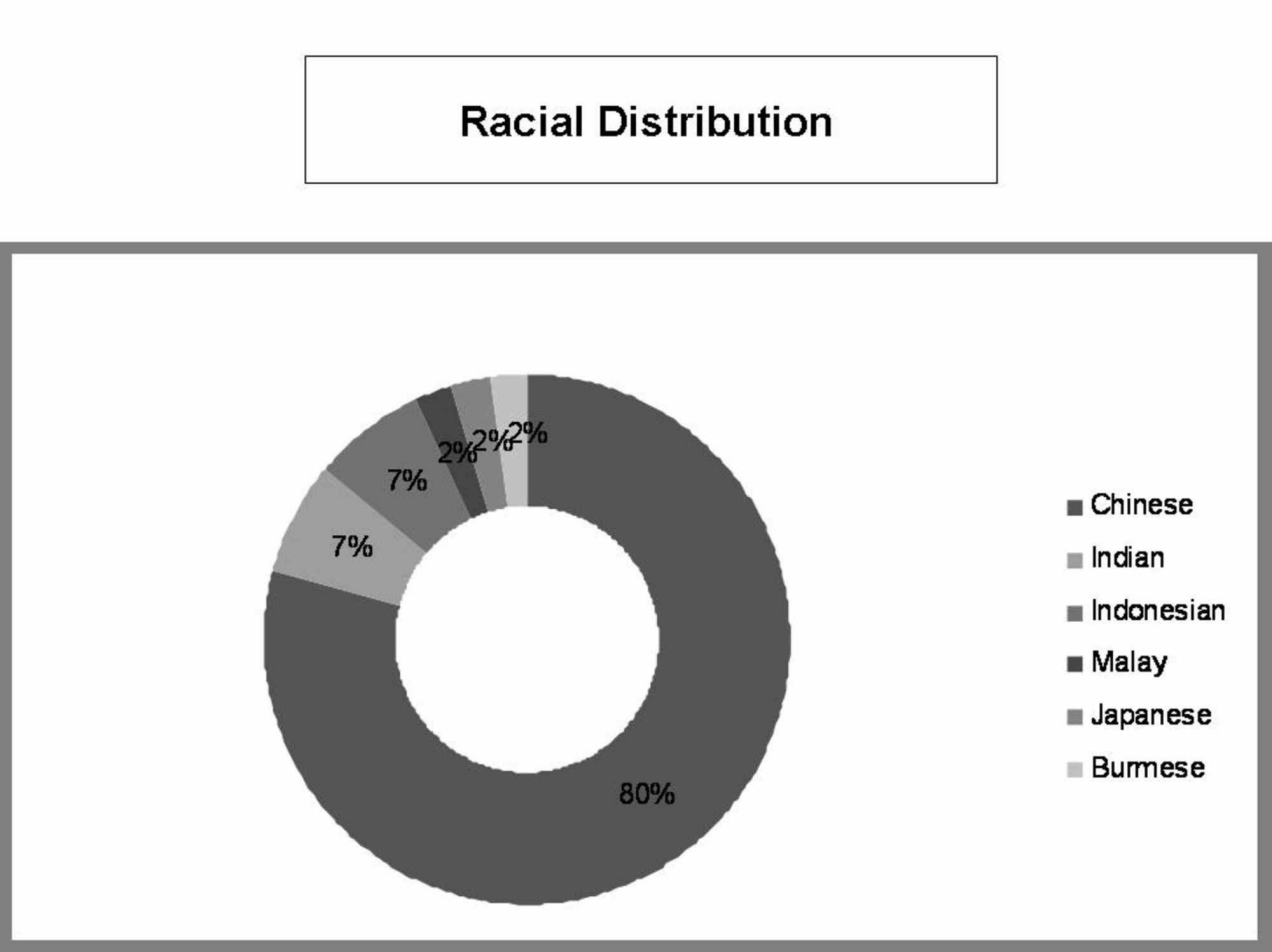
Racial distribution of all fungal rhinosinusitis (FRS) patients

The mean age of patients was 54 years, ranging from 21 to 79 years. There were 21 (47.7%) males and 23 (52.3%) females. All but one patient were Asians (97.7%) of which Chinese constituted the majority with a total of 34 patients (77.3%). Racial distribution is demonstrated in Figure 1.

Clinical presentation

Overall, 24 patients (54.5%) presented with symptoms of nasal blockage, congestion, and excess rhinorrhoea and/or post nasal drip, most of which (75%) were EMFRS, 18 (40.9%) with headache and localized facial pain that was more frequent in patients with FB (72.2%), 12 (27.3%) with epistaxis or bloody nasal discharge, and 14 (31.8%) with hyposmia/anosmia. Duration of symptoms from onset to presentation ranged between one and 120 months with an average of 16.6 months (Mean duration for EMFRS 23.7 months, FB 14.6 months). In four cases (9.1%), duration of symptoms has not been documented. Ten patients had multiple medical conditions including diabetes mellitus, hypertension, and ischemic heart disease with/without bronchial asthma. Nonetheless, their medical conditions were stable, under control, and all patients were immunocompetent.

Two FB patients presented with eye symptoms, one with gradual unilateral proptosis, visual loss, and ophthalmoplegia, and the second one with mild proptosis only. In both cases, the underlying cause was fungal ball with secondary acute complicated bacterial rhinosinusitis where no evidence for patient immune suppression, fungal tissue invasion, or histological features of granulomatous fungal infestation was detected. Another two cases of sphenoid sinus FB (16.7%) were incidentally discovered during CT scan that was performed by another department for unrelated complaints when both patients had no documented otorhinolaryngologic-related symptoms.

Amongst EMFRS patients, five gave history of previously diagnosed allergic rhinitis including fungal allergy and two others showed positive skin prick test (SPT) for fungal allergy yielding a total of seven cases (35%) with documented allergy to fungi. Four of the seven patients had all the other criteria for AFRS12 including presence of nasal polyposis, CT scan diagnostic features, allergic fungal mucin, and positive fungal stain within the mucin. Two of them (50%) gave history of associated bronchial asthma and multiple previous endoscopic sinus surgeries for recurrent similar disease. The rest of the three patients with documented fungal allergy had an associated bronchial asthma in addition to the other diagnostic criteria except for fungal stain which was not performed. As such, all the seven patients were regarded as allergic fungal rhinosinusitis. Deviated nasal septum (DNS) was seen in 14 cases (31.8%) of EMFRS, yet it was found to be unrelated to the side of predominant involvement.

Summary of clinical presentations for all, EMFRS, and FB patients is given in Tables 1-3.

**Table 1:** Clinical presentation of all patients with fungal rhinosinusitis (FRS) PND: Post-nasal drip

Clinical Presentation	Total (%)
Total number (%)	44 (8.26)
Males/Females (%)	21 (47.7)/23 (52.3)
Age (mean) years (%)	21-79 (54)
Duration of symptoms (%)	1-120 months (16.6)
Nasal blockage (%)	24 (54.5)
Rhinorrhea/PND* (%)	22 (50)
Pain/headache (%)	18 (40.9)
Epistaxis/bloody discharge (%)	12 (27.3)
Hyposmia/anosmia (%)	14 (31.8)
Eye symptoms (%)	2 (8.3)
Allergic rhinitis (%)	8 (18.2)
Mucosal edema/polyps (%)	26 (59.1)
Diagnostic CT* features (%)	44 (100)
Eosinophilic mucin/fungal debris	44 (100)
Positive fungal stain (%)	19 (43.2)
Positive fungal culture (%)	15 (34)
Follow-up range (mean) in months	1-60 (14.6)
Recurrence (%)	6 (13.6)

**Table 2:** Clinical presentation of patients with fungal ball PND: Post-nasal drip

Clinical Presentation	Fungal Ball
Total Number (%)	24 (4.5)
Males (%)/Females (%)	11 (55)/9 (45)
Age range (mean) in years	21-79 (51.7)
Mean duration of symptoms (months)	14.6
Nasal blockage (%)	8 (33)
Rhinorrhea/PND* (%)	4 (16.7)
Pain/headache (%)	13 (54.2)
Epistaxis/bloody discharge (%)	8 (33)
Hyposmia/anosmia (%)	4 (16.7)
Eye symptoms (%)	2 (8.3)
Secondary bacterial infection (%)	15 (62.5)
Incidental diagnosis (%)	2 (8.3)
Mucosal edema/nasal polyps (%)	7 (29.2)
Diagnostic CT* features (%)	24 (100)
Ball or mass of fungal debris (%)	24 (100)
Positive fungal stain (%)	15 (62.5)
Positive fungal culture (%)	11 (45.8)
Positive bacterial aerobic culture (%)	7 (29.2)
Diagnostic histology (%)	8 (33)
Follow-up range (mean) in months	1-34 (10.3)
Recurrence (%)	0 (0)

**Table 3:** Clinical presentation of patients with EMFRS EMFRS: Eosinophilic mucin fungal rhinosinusitis; PND: Post-nasal drip.

Clinical Presentation	EMFRS*	AFRS*	EFRS*
Total number (%)	20 (3.75)	7 (35)	13 (65)
Males (%)/Females (%)	10 (41.7)/14 (58.3)	3 (43)/4 (57)	8 (61.5)/5 (38.5)
Age range (mean) in years	21-79 (55.6)	29-75 (45)	21-76 (55.8)
Mean duration of symptoms (months)	23.7	1-24 (7.3)	1-120 (26.7)
Nasal blockage (%)	16 (80)	5 (71)	11 (85)
Rhinorrhea/PND* (%)	18 (90)	5 (71)	13 (100)
Pain/headache (%)	5 (25)	2 (28.6)	3 (23)
Epistaxis/bloody nasal discharge (%)	4 (20)	3 (43)	7 (53.8)
Hyposmia/anosmia (%)	10 (50)	1 (14.3)	2 (15.4)
Allergic rhinitis (%)	7 (35)	7 (100)	0
Bronchial asthma (%)	11 (55)	5 (71)	6 (46.2)
Nasal polyps (%)	19 (95)	7 (100)	12 (92)
Diagnostic CT* features (%)	20 (100)	7 (100)	13 (100)
Eosinophilic mucin/fungal debris (%)	20 (100)	7 (100)	13 (100)
Positive fungal stain/culture (%)	4 (20)	4 (57)	ND*
Follow-up range (mean) in months	21.4	1-60 (21.4)	1-60 (17.2)
Recurrence (%)	6 (30)	2 (28.6)	4 (30.8)

Computed tomography scan findings

**Figure 2:**
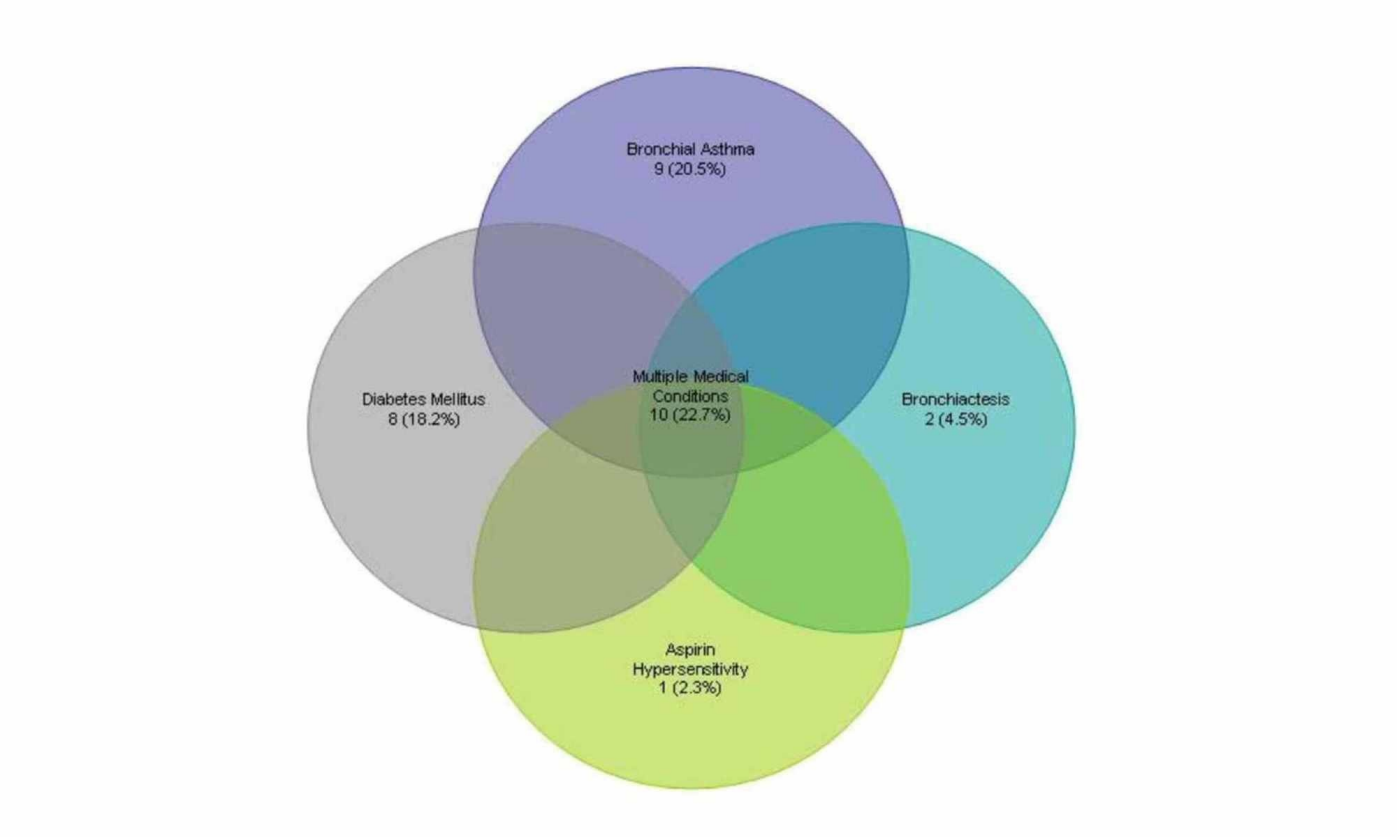
Prevalence of associated medical conditions among all fungal rhinosinusitis (FRS) patients

**Figure 3:**
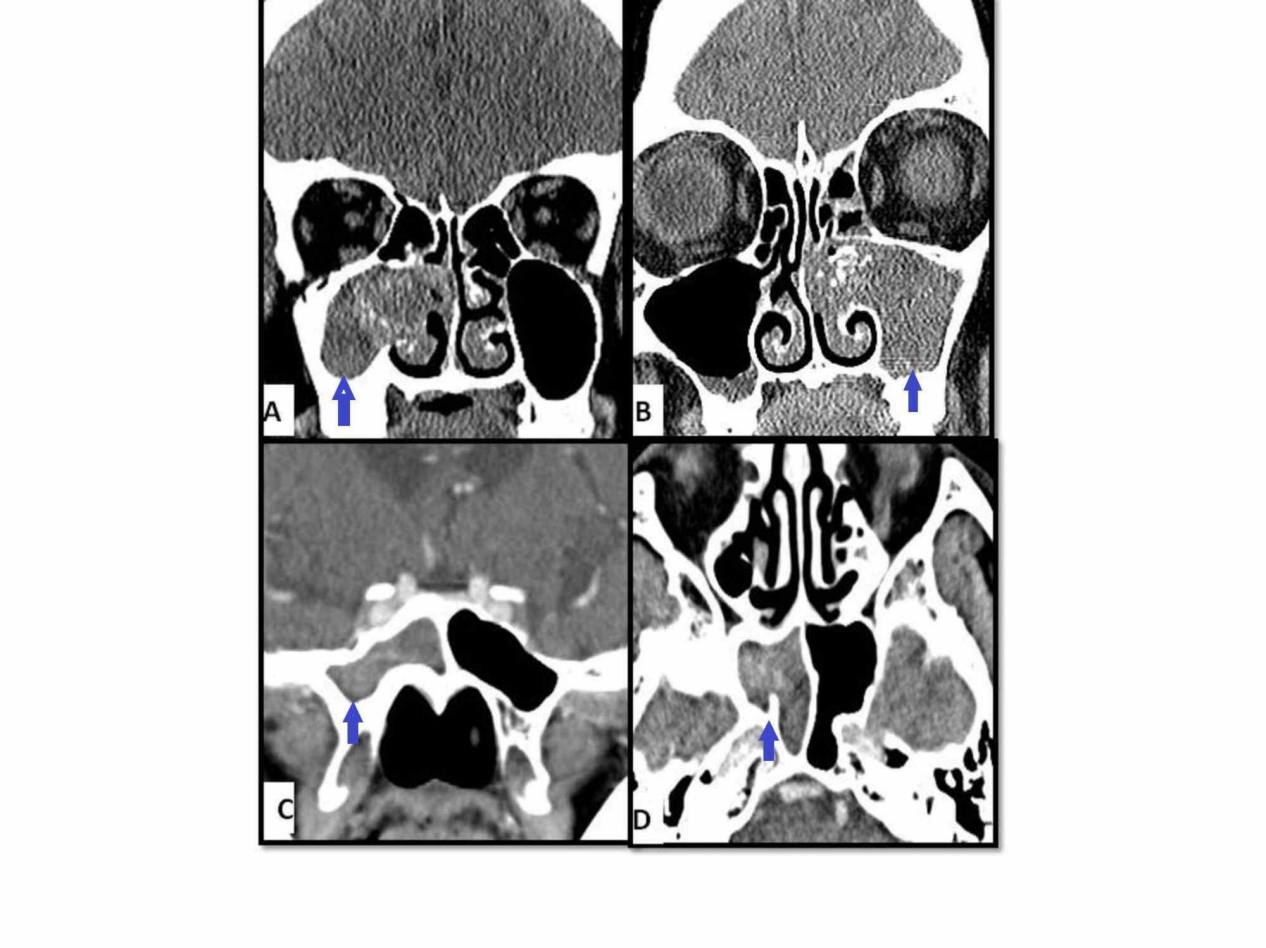
CT scans of fungal ball patients showing confined involvement of the right maxillary sinus (A), left maxillary sinus (B), and right sphenoid sinus (C & D)

CT scan showed hyperdense metallic shadows (calcification) in all cases of FRS. Bony remodeling (bony thinning/erosion/sinus expansion) was seen in eight cases (1 AFRS, 2 EFRS, and 5 FB). Lund-Mackay CT scan score for rhinosinusitis range was 2-13 for FB (mean = 6.2) and 8-23 for EMFRS (mean = 18.2) [13]. Figure 2 and Figure 3 show examples of CT scans for both FB and EMFRS patients.

Management

All patients of AFRS received pre-operative medical therapy in the form of oral antibiotic, intranasal steroid spray, and/or short courses of pulse systemic “oral” steroid therapy for variable periods of time ranging between one and three months. Among the FB patients, 15 were treated with antibiotics prior to surgery for clinical evidence of secondary bacterial rhinosinusitis and seven were given a short course of oral steroids pre-operatively to reduce tissue edema and inflammation. While ESS was the planned management for all cases of FB, for those with EMFRS, surgery was performed because of persistence of symptoms and/or signs despite adequate and maximum medical treatment.

**Figure 4:**
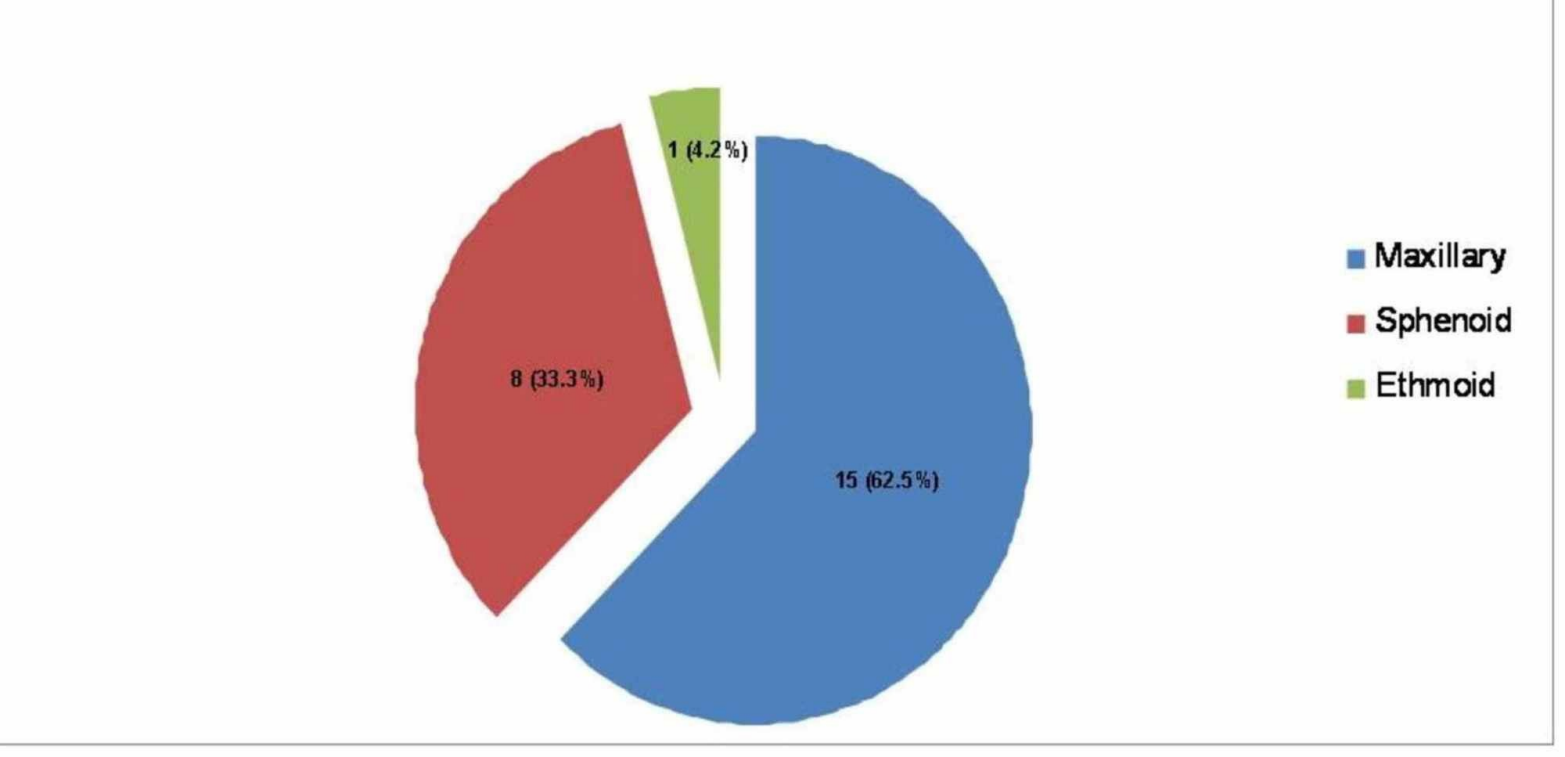
Distribution of fungal ball among the paranasal sinuses

All 44 patients have undergone ESS for disease clearance and ventilation of the sinuses. Involvement of all sinuses was noticed in all cases of AFRS, three of which were unilateral (15%). Single sinus involvement was observed in all cases of FBs. Maxillary sinus was involved in the majority of cases (62.5%). Distribution of sinus involvement by FB is seen in Figure 4.

There was no intra-operative documentation of any encountered bony dehiscence or erosion in all the cases.

All patients received a post-operative short course of oral antibiotic, analgesia, and normal saline nasal douches. In addition, all EMFRS patients received short course of low dose oral steroids for one to two weeks post-operatively, all continued on intranasal steroid spray, and one patient received oral itraconazole for a period of six months because of recurrent AFRS disease with history of three previous endoscopic sinus surgeries and a confirmative positive histology in the last operation at our institution. Duration of antifungal therapy was based on clinical evidence of disease clearance and endoscopic findings of healthy and normal looking sinus mucosa.

Among the FB cases, one patient received itraconazole for a period of one month because of suspicion of invasive fungal rhinosinusitis. This patient presented with orbital abscess secondary to an infected fungal ball of the sphenoid sinus and he had a history of diabetes mellitus which was otherwise under control. However, histopathology and fungal staining confirmed the diagnosis of fungal ball with no evidence for fungal invasion or granuloma formation.

Histopathology/fungal stain/culture

Histology reports of intra-operative specimens were available for 18 of 20 EMFRS patients; all showed features of chronic inflammatory changes including stromal oedema with marked predominant eosinophilic and minimal lymphoplasmocytic and neutrophilic infiltration. Out of nine cases where fungal staining was used (Gomori-Grocott methenamine silver "GMS" and Periodic-acid Schiff "PAS" stains), four were positive and were culture positive as well.

**Figure 5:**
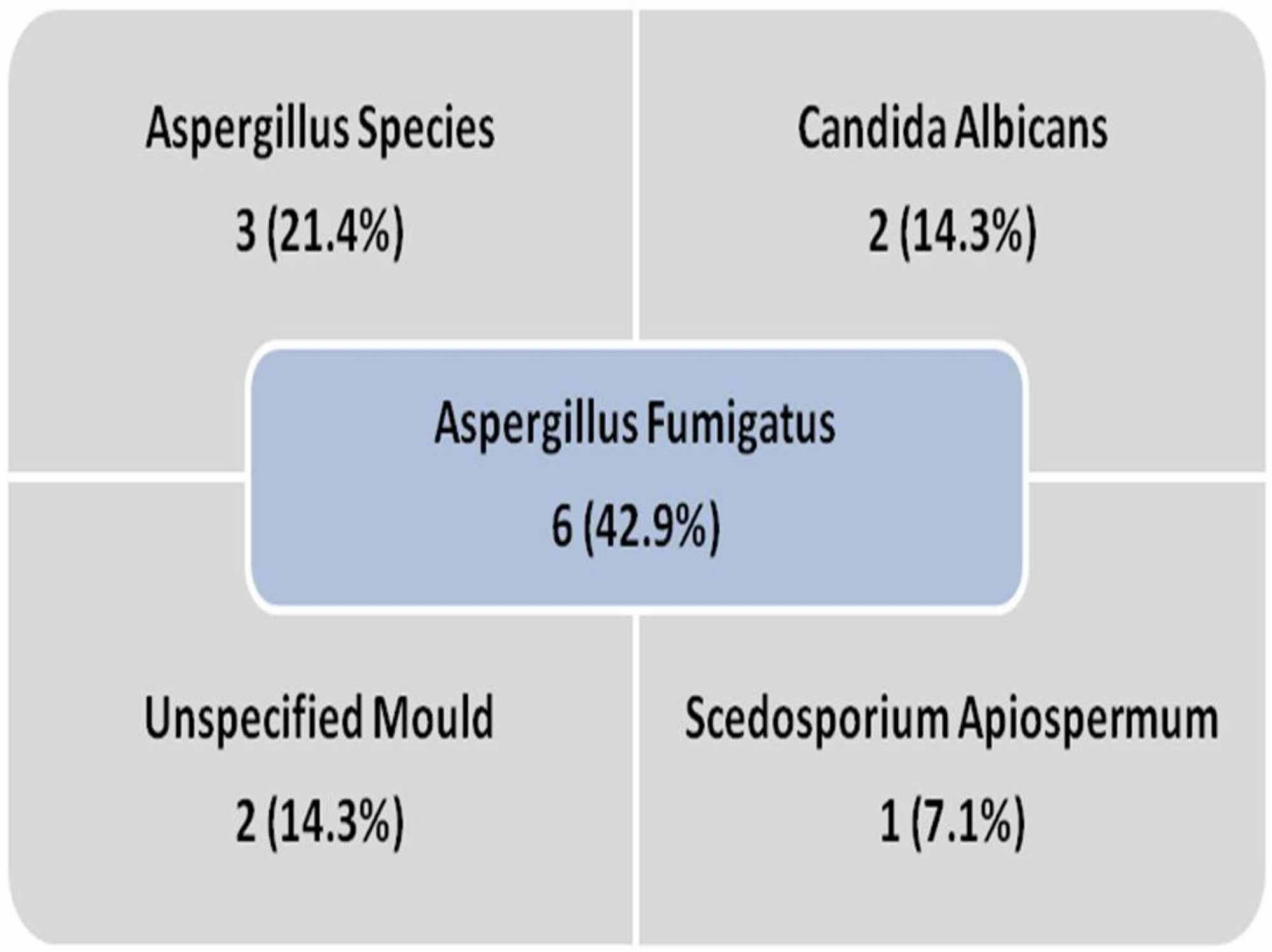
Results of fungal culture

In the fungal ball group, eight specimens were reported as dense masses of impacted fungal spores and hyphae in an onion skin-like appearance confirming the diagnosis of FB, 11 cases were reported as chronic inflammation based on tissue specimens alone without fungal debris, and five cases lack histology specimens. Fungal staining and culture was done for 23 cases of which 11 were positive. Aspergillus species were the commonest fungi isolated amongst all cases. Results of fungal cultures are summarized in Figure 5.

Follow-up and recurrence

Follow-up period ranged between one and 60 months with a mean of 14.6 months (EMFRS 21.4, FB 10.3). Recurrence was encountered seven times in six of EMFRS patients (30%) at three to 24 months post-operatively, yet only one (16.7%) required revision endoscopic sinus surgery after failed medical treatment. The rest responded very well to maximum medical therapy with oral antibiotic, short course of oral steroids, and long-term intranasal steroid spray. No recurrence or residual disease was seen in any FB patients (Tables 1-3).

## Discussion

Chronic rhinosinusitis (CRS) is an inflammatory disorder of the mucosal lining of the nose and paranasal sinuses with numerous predisposing factors, including genetics, anatomic anomalies, allergy, bacteria, and fungus [13,14]. Fungal rhinosinusitis is not an uncommon form of rhinosinusitis. It is estimated that fungal rhinosinusitis is encountered in about 10% of surgically treated CRS [15]. In some studies, using highly sensitive culture techniques, fungi have been isolated from nearly 100% of patients with rhinosinusitis and nasal polyposis. However, similar findings were obtained from healthy control individuals making the role of fungus in CRS further complicated [16]. The spectrum of conditions included under the diagnosis of FRS includes AFRS, FB, SFI, AIFRS, CIFR, and GFRS [17]. Differentiation between each subtype is based on clinical presentation, histological features, and to some extent the immunological status of the patient which may play a role in pathogenesis [7]. While non-invasive types of FRS are usually seen in otherwise healthy and immunologically competent individuals, this does not seem to be the role in invasive FRS. Acute invasive FRS usually affects immunologically suppressed patients such as those with diabetes mellitus, acquired immunodeficiency syndrome, leukemia, or those on long-term immunosuppressive medications [18]. On the other hand, chronic invasive and granulomatous FRS could be seen in either population [7].

There is a major controversy in the literature surrounding the categorization of allergic FRS [19]. Some investigators suggested that FRS in which abundant eosinophils are seen on histological examination should be regarded as eosinophilic FRS (EFRS) and that could be further categorized into either allergic or non-allergic EFRS depending on whether or not IgE-mediated fungal allergy is identified [20].

The significance fungus contributing to the development of rhinosinusitis remains unknown. Despite the fact that fungus growth is more often seen in hot and humid (tropical) climatic regions, in our series, only 8.3% of all surgically treated CRS patients were FRS. In a report from south India, FRS was histologically diagnosed in 45.7% of surgically treated cases where tissue specimens were sent for examination [21].

Although variations in the standards and definitions for diagnosing AFRS exist, current estimated incidence among CRS cases that undergo surgery ranges between 5% and 12% [1,4,8]. In this series, the incidence of AFRS was 3.8% which is slightly less than internationally recognized figures. In a study conducted in Singapore, allergy to different fungi species was identified in 26 to 32% of asthma and/or allergic rhinitis patients [22]. However, not all patients with allergy to fungi eventually develop AFR. Thus, further studies are needed in order to determine the underlying risk factors and the exact pathophysiology of the disease.

The diagnosis of AFRS in this study is based on Bent and Kuhn proposed criteria which include the identification of IgE mediated allergy to fungi, presence of CRS with nasal polyposis, characteristic CT scan features, presence of eosinophilic mucin, and positive identification of fungus either by special stains or by culture [23]. However, since not all of patients have documented atopy nor histological finding of fungus, other added criteria were used to assess in reaching the correct diagnosis including positive fungal culture, presence of asthma, diffuse involvement of the sinuses, and tendency for recurrence.

Patients with AFRS are usually young adults, who are immunocompetent, atopic, and tend to present clinically as CRS with nasal polyposis and eosinophilic mucin which frequently involves all sinuses, but could present as unilateral disease during early stage, and may occasionally present with features of remodeling and expansion of the facial bones such as proptosis and widening of the nasal bridge indicating long standing disease [11]. Mean age at presentation is typically in the mid twenties, yet in the current series, the mean age was much older at 54 years ranging between 21 and 79 years [24]. This could be attributed to the fact that some of our patients have received previous medical treatment and surgeries in other hospitals before the current presentation to our hospital with lack of medical data about their previous sinonasal condition. In general, AFRS affects predominantly females, a finding that is consistent with this current report [11,25].

Radiologically, FB appears as an expansile hyperattenuating mass with a classic punctuate calcifications that is usually localized to one sinus. On MRI, however, FB is hypointense on T1-weighted and T2-weighted images with hyperintense sinus mucosal lining in contrast enhanced T2-weighted images owing to local tissue inflammation [26]. Such features were observed in all FB cases of this report. Maxillary sinus is the most commonly involved by FB, followed by sphenoid and then ethmoid sinuses. Frontal sinus FB is extremely rare [15,26]. The result of this study is consistent with the literature, with maxillary sinus most common being involved in two-thirds of cases, sphenoid sinus in one-third, and ethmoid sinus in only one case. Macroscopically, FB appears as dense mass within the sinus cavity made of friable cheesy-like material that is green, yellow, brown or black and easily peeled off the sinus mucosa [27].

Saprophytic fungal infestation among surgically treated CRS has not been encountered in this series. This is probably due to the fact that saprophytic fungi usually present on nasal secretions or crusts of previously operated patients in which nasal toilet and cleaning of the widely opened sinus cavities at the outpatient clinic is all what is required to manage such presentation. In addition, invasive forms of FRS whether acute, chronic, or granulomatous types were also not encountered.

## Conclusions

The incidence and prevalence of different forms of fungal rhinosinusitis are increasing world wide, likewise the diversity of the pathogenic fungi. With advances in both endoscopic surgery and imaging technologies, diagnosis and management of different types of fungal rhinosinusitis have been better defined. In Singapore, the most common type of FRS is FB, followed by AFRS. Despite the lack of evidence for IFRS in this series, high index of suspicion and vigilance to exclude invasive fungal rhinosinusitis especially when dealing with cases of complicated sinusitis in immunocompromised individuals is strongly emphasized.
